# How sagittal alignment changes can affect independent horizontal gaze after neuromuscular scoliosis correction

**DOI:** 10.1186/s13018-025-06089-0

**Published:** 2025-07-15

**Authors:** Sung Taeck Kim, Hyoungmin Kim, Bong-Soon Chang, Seonpyo Jang, Junyeop Lee, Sam Yeol Chang

**Affiliations:** Department of Orthopedic Surgery, Seoul National University Hospital, 101 Daehak-ro, Jongno-gu, Seoul, 03080 Korea

**Keywords:** Neuromuscular scoliosis, Horizontal gaze, Sagittal alignment

## Abstract

**Background:**

When surgical correction for spinal deformity is performed for neuromuscular scoliosis (NMS) patients, sudden changes in sagittal spinal alignment after surgery can cause difficulties in maintaining independent horizontal gaze. Typically, patients with weak neck flexors can experience a loss of head control during neck extension that cannot be restored to a neutral position to allow horizontal gaze. This study aimed to analyze the radiological factors associated with the ability to maintain an independent horizontal gaze following deformity correction surgery in patients with NMS.

**Materials and methods:**

Patients who underwent deformity correction surgery for the NMS from 2013 to 2023 were included. We assessed whether the patient could maintain a horizontal gaze without a headrest after deformity correction. Clinical demographics and radiographic alignment parameters before and after surgery were collected. Multivariable logistic regression was performed to identify risk factors of postoperative loss of head control.

**Results:**

88 NMS patients were included in this study. After deformity correction, 31 (35%) patients could not maintain a horizontal gaze without a headrest at postoperative 3 months. The group of patients who maintained horizontal gaze had a significantly greater postoperative chin-brow vertical angle (4.3°±-10.6 vs. -4.7°±11.6, *p* = 0.003) and C7-S1 sagittal vertical axis (67.0 mm ± 46.5 vs. 29.3 mm ± 40.1, *p* = 0.002) than the group who could not. Logistic regression analysis showed that a greater increase of lumbar lordosis (odds ratio = 1.018, *p* = 0.039) and change in C7-S1 sagittal vertical axis (odds ratio = 1.020, *p* = 0.025) were associated with an inability to maintain horizontal gaze.

**Conclusions:**

In this study, the loss of ability to maintain independent horizontal gaze after deformity correction for NMS was associated with a greater increase in lumbar lordosis. Maintaining preoperative sagittal balance is crucial for head control in patients undergoing deformity correction for NMS.

## Introduction

Neuromuscular scoliosis (NMS) can occur in patients with neuromuscular diseases (NMD). The progression of muscle weakness results in changes to trunk stability, eventually leading to progressive scoliosis [1, 2]. In the coronal plane, the development of a long collapsing C-shaped scoliosis, which continues down to the pelvis, leads to marked pelvic obliquity and elevation of the iliac crest on the concave side of the curvature [3]. Progressive NMS can impair the ability of a non-ambulatory patient to sit comfortably in a wheelchair and perform activities of daily living [4, 5]. 

In the sagittal plane, patients with NMD can experience problems with neck movement and head control as the disease progresses [6]. Severe weakness of the neck extensor muscles resulting in “dropped head syndrome” often occurs in patients with NMD with a natural course [7]. The dropped head syndrome is characterized by a chin-on-chest deformity in the sitting position that can be corrected with passive neck extension. The dropped head syndrome is a phenomenon that makes horizontal gaze difficult in patients with NMD due to weakened neck extensor muscles.

**Fig. 1:**
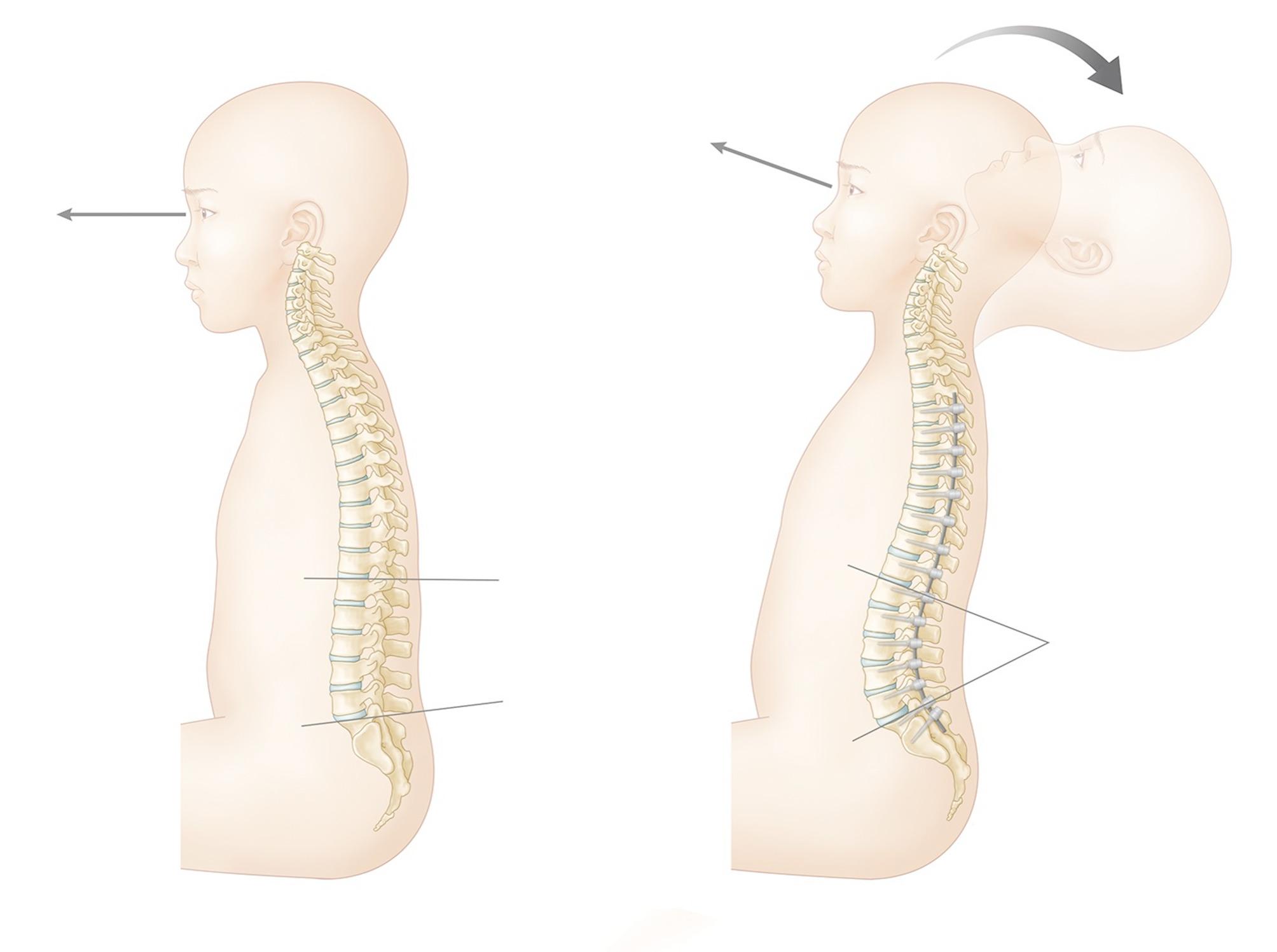
Preoperative maintenance of independent horizontal gaze (left) and postoperative inability to maintain horizontal gaze due to relative neck flexor weakness after deformity correction (right)

In contrast to dropped head syndrome and chin-on-chest deformity, the authors have observed patients with NMD who experience loss of head control in the opposite direction after deformity correction for NMS [8]. These patients, who were able to control their neck movement before surgery, lose their ability to flex their neck and maintain horizontal gaze after deformity correction (Fig. 1). Typically, their heads fall posteriorly during neck extension and cannot return to neutral against gravity due to neck flexor weakness. In these patients, using a headrest becomes inevitable to maintain horizontal gaze.

Biomechanical studies support the clinical observation that the cervical flexor musculature may be disadvantaged in such scenarios. In the study by Vasavada et al., even in healthy individuals, the maximal isometric strength of the neck flexor muscles was reported to be only approximately 70% of that of the extensor muscles, indicating an inherent biomechanical disadvantage in resisting anterior head flexion forces [9]. This suggests the flexor group may be more vulnerable to strength deterioration in patients with neuromuscular disorders.

In addition, Lim et al. demonstrated that individuals with loss of cervical lordosis exhibited significantly reduced baseline activation in the upper trapezius and rhomboid muscles across various static and dynamic postures [10]. Interestingly, despite the low resting activity, the upper trapezius showed paradoxically increased relative activation during neck flexion as a compensatory response to the increased bending moment generated by anterior displacement of the head’s center of gravity. These findings further suggest that altered sagittal alignment significantly impacts the biomechanical demands on cervical musculature.

However, studies regarding postoperative loss of head control due to relative neck flexor weakness are lacking. Thus, this study aimed to identify the risk factors for postoperative loss of head control after spinal deformity correction surgery for NMS by analyzing demographic, physical, radiological, and surgical aspects.

## Materials and methods

### Study design and data collection

This study was approved by the Institutional Review Board of our institution (2208-012-1346). In this study, we retrospectively reviewed consecutive patients who underwent posterior spinal deformity correction using a pedicle screw fixation system for NMS from January 2013 to December 2023 in a single tertiary hospital.

We only included wheelchair-dependent NMD patients who could sit independently, controlled their neck movement, and did not require a headrest before surgery. Furthermore, all patients had anatomically located hips without evidence of dislocation or subluxation. Patients with a history of previous spine surgery and inadequate radiographic examinations, including whole spine lateral radiographs in a sitting position, were excluded from the study.

Demographic data and the type of NMD before surgery were collected. As for the radiological sagittal alignment parameters, cervical lordosis (CL), thoracic kyphosis (TK, T5-T12), lumbar lordosis (LL), C2-C7 sagittal vertical axis (SVA), and C7-S1 SVA were measured from whole-spine lateral radiographs. As indicators of head posture, T1 slope (T1S) and chin-brow vertical angle (CBVA) were also measured [11]. We also assessed pelvic obliquity angle using the Osebold technique from whole-spine anteroposterior radiographs in a sitting position [12]. These radiographic parameters were measured from radiographs taken one day before surgery and three months postoperatively.

The primary outcome of this study was the patient’s ability to control their head by active neck flexion and maintain horizontal gaze after surgery. Based on the standardized physical assessment during routine clinical evaluation, patients were classified into the headrest required group if they could not keep a horizontal gaze while sitting upright at 3 months postoperatively, Those who could not actively bring his head to a neutral position without any assistance after full neck extension were also included in the headrest required group. Otherwise, patients were classified as the headrest non-required group.

### Statistical analysis

Statistical analysis was performed using IBM (Armonk, NY, USA) SPSS Statics software version 28.0. Descriptive statistics were applied to summarize demographic and clinical characteristics. Categorical variables were analyzed using the chi-square test. The distribution of continuous variables was assessed using the Shapiro–Wilk test to evaluate normality. For variables that followed a normal distribution, values were reported as mean ± standard deviation (SD), and comparisons between groups were performed using the Student’s t-test. For variables that did not conform to a normal distribution, values were expressed as median with interquartile range (IQR: Q1–Q3), and the Wilcoxon signed-rank test was employed accordingly. Variables found to be non-normally distributed based on the Shapiro–Wilk test included preoperative cervical lordosis, thoracic kyphosis, lumbar lordosis, C7–S1 SVA, and postoperative C2–C7 and C7–S1 SVA.

As for the logistic regression analyses, covariates for multivariable logistic regression were selected based on univariate analysis results (*p* < 0.05) to ensure the inclusion of potentially relevant predictors. Multicollinearity among variables was assessed using the variance inflation factor (VIF), with values > 5 indicating significant collinearity. Variables with high collinearity were excluded to improve model stability. No formal adjustment for multiple comparisons was applied, given the limited number of predictors and the hypothesis-driven nature of the model. The predictive value of lumbar lordosis correction angle and ∆C7–S1 SVA was evaluated using joint receiver operating characteristic (ROC) curves.

## Results

Among 104 wheelchair-bound patients who underwent posterior spinal deformity correction surgery from NMS during the study period, five were excluded due to the absence of sitting whole spine lateral radiographs, and 11 were excluded due to preexisting problems with head control. As a result, 88 patients with a mean age of 13.9 ± 2.4 (male 69, female 19) were included in this study.

In this study, 31 (35%) patients showed loss of head control at 3 months postoperatively, requiring a headrest to maintain horizontal gaze. There was no statistically significant difference in demographics and preoperative diagnosis between the patients who maintained or lost their head control ability (Table 1). However, the headrest-required group tends to have a higher proportion of girls than the non-required group (32.2% vs. 15.8%, *p* = 0.128).

There were no significant differences between the two groups in the preoperative CL, TK, LL, CBVA, T1S, C2–C7 SVA, C7–S1 SVA, CBVA, and pelvic obliquity (Table 2). However, the postoperative CBVA (-4.7 ± 11.6° vs. 4.3 ± 10.6°, *p* = 0.003) and T1 slope (2.0 ± 12.7° vs. 16.8 ± 11.9°, *p* < 0.001) was significantly smaller in the headrest-required group. The headrest-required group also showed significantly greater change in C2-C7 SVA (*p* = 0.004) and C7–S1 SVA (*p* = 0.002).

When the amount of change in sagittal alignment parameters before and after surgery was compared between the two groups, the two groups showed no significant difference in CL and TK (Table 3). However, the change in LL was larger in the headrest-required group at postoperative 3 months (36.1 ± 11.9° vs. 29.3 ± 21.2 °, *p* = 0.006) (Table 3).

Risk factors for postoperative loss of head control found using logistic regression analysis were LL correction angle (OR = 1.018 [95% CI 1.001–1.036], *p* = 0.039), postoperative CBVA (OR = 0.917 [95% CI 0.857–0.982], *p* = 0.012), and ∆C7-S1 SVA (OR = 1.020 [95% CI 1.004–1.051], *p* = 0.025) (Table 4). These results indicate that, as the LL correction angle and ∆C7-S1 SVA increased by 1 °or 1 mm, the risk of postoperative loss of head control increased by 1.8% and 2.0%, respectively. Furthermore, postoperative CBVA increased by 1 °, and the risk of postoperative loss of head control decreased by 8.3%.

**Fig. 2:**
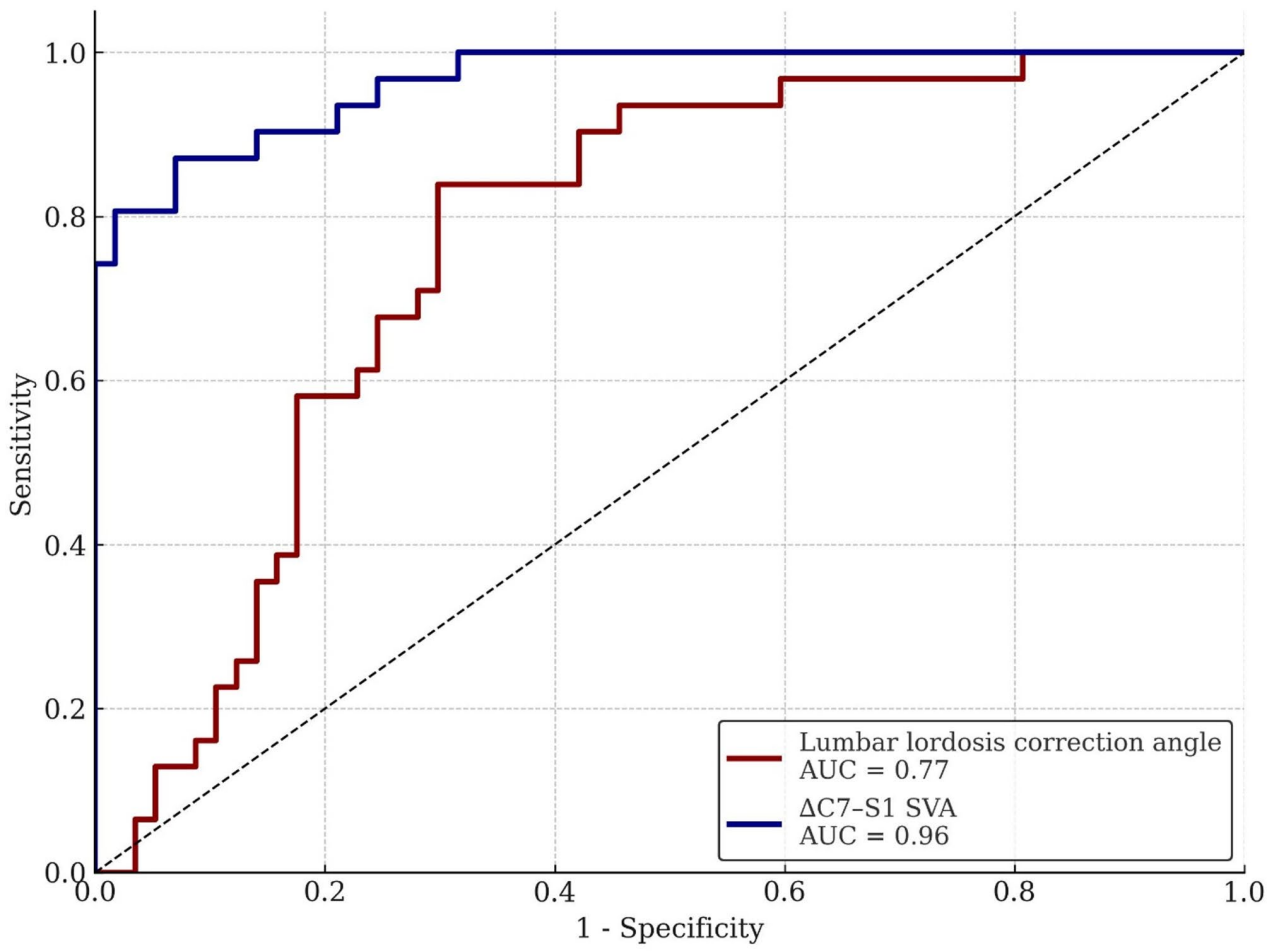
Receiver operating characteristic (ROC) curves for Lumbar lordosis correction angle and ∆ C7–S1 SVA were calculated for all patients included in this study. AUC, area under curve, SVA, sagittal vertical axis

Receiver operating characteristic (ROC) curve analysis was performed to evaluate the predictive value of LL correction angle and ∆C7–S1 sagittal vertical axis (SVA) for postoperative headrest dependence. The area under the curve (AUC) was 0.77 (95% CI, 0.66–0.86; *p* < 0.001) for the LL correction angle and 0.91 (95% CI, 0.83–0.97; *p* < 0.001) for ∆C7–S1 SVA, indicating good to excellent discriminatory ability (Fig. 2). The optimal cut-off value for predicting postoperative headrest requirement was 33.7° for lumbar lordosis correction angle (sensitivity 0.84, specificity 0.70) and 28.4 mm for ∆C7–S1 SVA (sensitivity 0.87, specificity 0.82), as summarized in Table 5.

**Table 1:** Demographic and clinical characteristics of patients based on postoperative loss of head control

	Headrest required group (*N* = 31)	Headrest non-required group (*N* = 57)			*P*-value
Age at surgery (year)	13.7 ± 3.0		13.9 ± 2.1		0.696
Sex					0.128
Male	21		48		
Female	10		9		
Height (cm)	147.6 ± 13.2		153.7 ± 11.1		0.271
Weight (kg)	39.2 ± 15.8		43.4 ± 14.4		0.224
Body mass index (kg/m^2^)	17.6 ± 5.3		18.4 ± 5.8		0.684
Diagnosis					0.922
Congenital muscular dystrophy	6			13	
Duchenne muscular dystrophy	13			23	
Spinal muscular atrophy	7			14	
Other*	5			7	

*Other: cerebral palsy (8), limb-girdle muscular dystrophy (3), and Leigh disease (1)

**Table 2:** Radiographic parameters between the headrest required group and the headrest non-required group

	Headrest required group (*N* = 31)	Headrest non-required group (*N* = 57)			*P*-value
**Preoperative**					
Cervical lordosis (°) §	10.6 (1.16, 16.02)		13.3 (3.16, 25.94)		0.435ª
Thoracic kyphosis (°) §	6.7 (-4.55, 18.87)		13.6 (6.79, 25.51)		0.291ª
Lumbar lordosis (°) §	0.4 (-42.42, 22.80)		-6.5 (-12.38, 25.92)		0.478ª
Chin-brow vertical angle (°)	5.2 ± 9.4		5.4 ± 9.0		0.935
T1 slope (°)	16.9 ± 16.2		21.8 ± 10.8		0.173
C2-C7 SVA (mm)	11.3 ± 21.9		20.5 ± 19.1		0.087
C7-S1 SVA (mm) §	79.9 (16.74, 95.14)	88.5 (53.60, 99.39)			0.531ª
Pelvic obliquity angle (°)	30.8 ± 16.2			27.4 ± 15.6	0.338
**Postoperative**					
Cervical lordosis (°)	4.2 ± 17.5			10.1 ± 19.3	0.217
Thoracic kyphosis (°)	11.9 ± 16.8			17.1 ± 17.8	0.152
Lumbar lordosis (°)	27.6 ± 18.2			28.9 ± 13.9	0.741
Chin-brow vertical angle (°)	-4.7 ± 11.6			4.3 ± 10.6	0.003*
T1 slope (°)	2.0 ± 12.7			16.8 ± 11.9	< 0.001*
C2–C7 SVA (mm) §	1.1 (-7.19, 9.82)			16.6 ± 20.2	0.004ª*
C7–S1 SVA (mm) § Pelvic obliquity angle (°)	29.3 (-8.30, 52.57) 9.9 ± 8.4			67.0 (51.31, 91.41) 11.3 ± 8.5	0.002ª* 0.461

SVA, sagittal vertical axis

§ The data in the table are represented as median (Q1, Q3) ª Wilcoxon signed-rank test was chosen

**Table 3:** Association between differences in values pre- and postoperative radiographic parameters and postoperative loss of head control

	Headrest required group (*N* = 31)			Headrest non-required group (*N* = 57)	*P*-value
**∆** Cervical lordosis (°)		-5.6 ± 8.3	-5.0 ± 6.1		0.801
**∆** Thoracic kyphosis (°)		5.9 ± 11.8	8.3 ± 6.3		0.298
**∆** Lumbar lordosis (°)		36.1 ± 11.9	29.3 ± 21.2		0.006*

**Table 4:** Multivariable logistic regression analysis for postoperative loss of head control

	Coefficient (β)		Adjusted OR		95% CI		VIF	*P* value
Lumbar lordosis correction angle (°)	0.017	1.018				1.001–1.036	2.89	0.039*
Postoperative CBVA (°)	-0.086	0.917				0.857–0.982	3.12	0.012*
∆ T1 slope (°)	0.026	1.027				0.973–1.084	2.54	0.314
∆ C2–C7 SVA (mm)	0.030	1.031				0.998–1.063	3.16	0.235
∆ C7–S1 SVA (mm)	0.019	1.020				1.004–1.051	1.35	0.025*

CBVA, chin-brow vertical angle, SVA, sagittal vertical axis, VIF, variance inflation factor

VIF is used to detect multicollinearity. A VIF < 5 indicates no significant collinearity issues

**Table 5:** AUC and cut-off

	Cut-off value		Sensitivity	Specificity	AUC	95% CI	*P* value
Lumbar lordosis correction angle (°)		33.7	0.84	0.70	0.77	0.66–0.86	< 0.001
∆ C7–S1 SVA (mm)		28.4	0.87	0.82	0.91	0.83–0.97	< 0.001

SVA, sagittal vertical axis

## Discussion

The goal of deformity correction in patients with NMS is to maintain independent sitting balance and participation in daily activities such as interacting with others, eating meals, watching television, and reading books [13, 14]. To achieve this goal, head control and horizontal gaze are essential. However, the authors have observed that while some patients achieve improved sitting balance in the coronal plane, head control problems in the sagittal plane can occur after deformity correction [8]. According to the previous study, in patients with cerebral palsy, the quality of preoperative trunk control has been reported to influence postoperative head control [15]. Giannini et al. reported that rigid neck hyperextension and poor head control are associated with the progression of Duchenne muscular dystrophy (DMD) [16]. Still, the risk factor of loss of head control after surgery has not been documented in the literature.

**Fig. 3:**
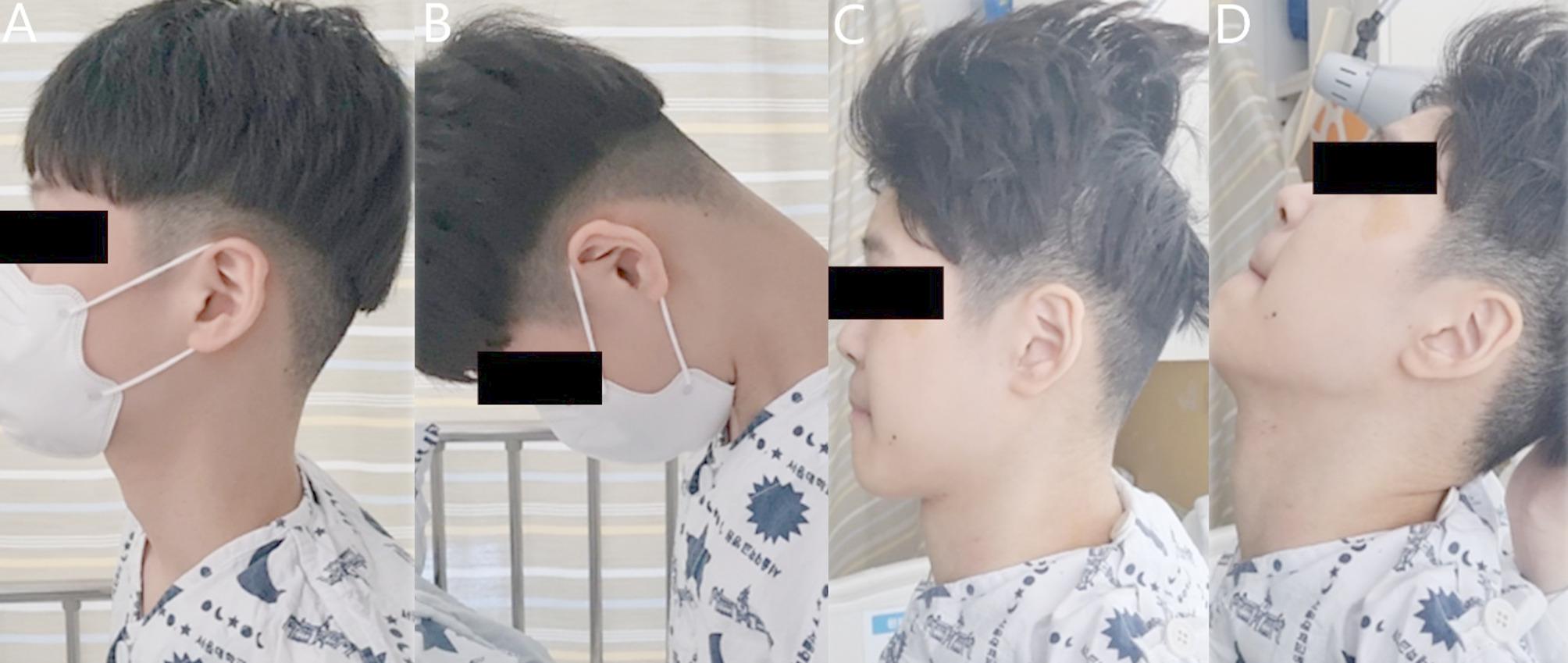
A representative case where the patient lost head control ability after NMS correction. Before surgery, the patient could return his head to a neutral position (**A**) by active neck flexion after full neck extension (**B**). However, after NMS correction, the patient could not return his head to a neutral position (**C**) after full neck extension (**D**)

Fig. 3 describes a case in the headrest-required group where the loss of head control (inability to flex his neck and maintain independent horizontal gaze) occurred after deformity correction for NMS. Before surgery, the patient was able to control his head movement, actively flex his neck, and put his head in a neutral position following full neck extension (Fig. 3A and B). However, after deformity correction, the patient could not flex his neck after full neck extension without any assistance, requiring a headrest to prevent irreversible neck extension (Fig. 3C and D).

The current study aimed to identify clinical and radiological factors associated with loss of head control after NMS correction. In this study, we found significant differences in postoperative CBVA, T1S, C2-7 SVA, and C7-S1 SVA values between the headrest-required group and the non-required group. When we focus on the changes in the sagittal alignment parameters before and after surgery, the headrest required group showed a significantly greater LL correction angle. In addition, multivariable regression analysis suggested that LL correction angle and ∆C7-S1 SVA exhibited significantly higher odds of postoperative loss of head control.

Among these parameters, the chin-brow vertical angle (CBVA) is a critical parameter for ensuring optimal horizontal gaze and was initially described in patients with ankylosing spondylitis [17]. Song et al. reported that the optimal chin-brow vertical angle (CBVA) in ankylosing spondylitis kyphosis is 10–20° [18]. However, no previous studies have investigated the appropriate CBVA values for patients with NMS, particularly those with weakened neck muscle power. In this study, we observed that a smaller CBVA is associated with an increased risk of impaired head control in maintaining horizontal gaze among patients with NMS.

Decreased LL and positive sagittal balance are the main concerns in ambulatory patients with adult spinal deformity (ASD) [19]. The restoration of ideal LL and correction of preoperative sagittal imbalance, which can lead to improvements in horizontal gaze, are the main goals of ASD surgery [20]. However, the clinical significance of LL and positive sagittal balance in maintaining a horizontal gaze is different in non-ambulatory NMS patients. In daily life, most NMS patients have decreased LL and positive sagittal balance due to core muscle weakness. These patients with positive sagittal balance maintain their horizontal gaze by extending their neck using relatively stronger neck extensor muscles. However, when LL is significantly restored, and positive sagittal balance is corrected by deformity correction surgery, relatively weak neck flexors fail to hold patients’ heads in neutral positions (Fig. 1).

**Fig. 4:**
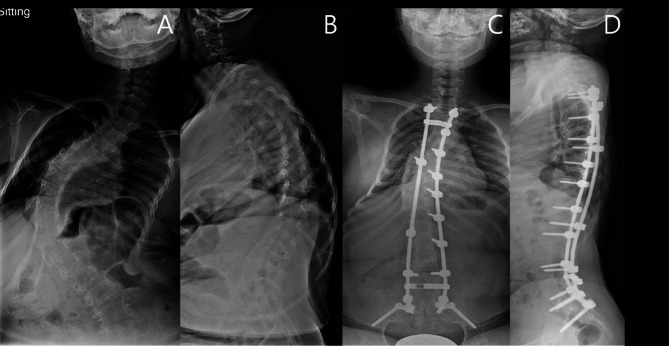
Whole-spine radiographs of a patient in the headrest required group. Preoperative (**A**) anterior-posterior view, (**B**) lateral view; postoperative (**C**) anterior-posterior view, and (**D**) lateral view

**Fig. 5:**
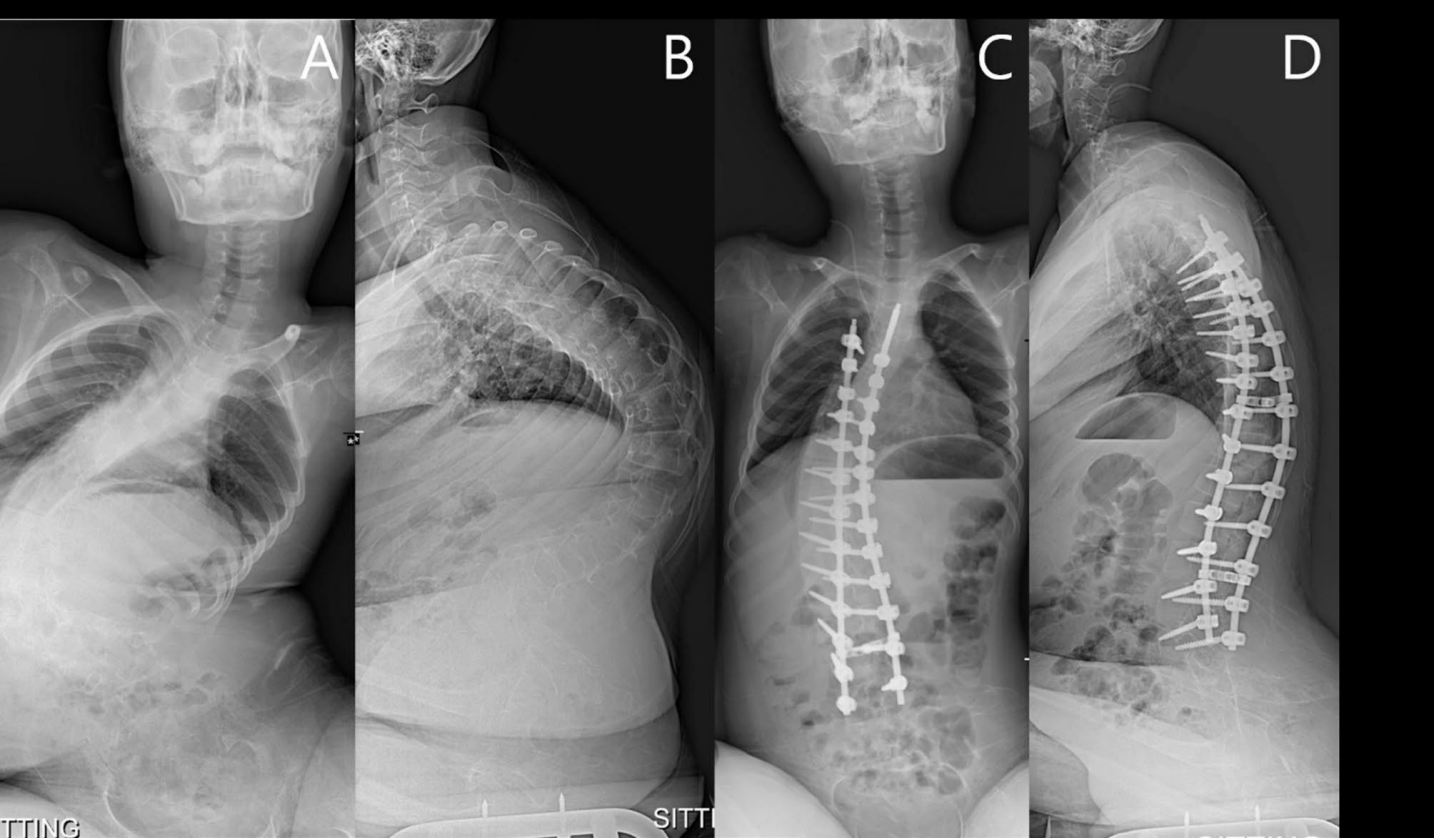
Whole-spine radiographs of a patient in the headrest non-required group. Preoperative (**A**) anterior-posterior view, (**B**) lateral view; postoperative (**C**) anterior-posterior view, and (**D**) lateral view

Figs. 4 and 5 are case examples of different outcomes of head control after deformity correction in NMS patients. The patient presented in Fig. 4 exhibited markedly increased LL and correction of sagittal balance after surgery. However, the patient experienced a loss of head control and the ability to maintain an independent horizontal gaze. In contrast, the patient shown in Fig. 5 displayed a minimal increase in LL and appropriate sagittal balance correction, which preserved head control and the ability to maintain an independent horizontal gaze. While cases like Fig. 4 may appear to have better radiographic sagittal alignment and balance, clinical outcomes related to head control and horizontal gaze are superior in cases like Fig. 5.

Our ROC curve analysis identified an LL correction angle of 33.7° as the optimal threshold for predicting postoperative headrest dependence. Based on this finding, we suggest that surgeons consider limiting the extent of LL correction to less than 33.7° during surgical planning, particularly in patients at risk, to help prevent postoperative loss of head control. While ∆C7–S1 SVA showed superior predictive value on ROC analysis (AUC: 0.91 vs. 0.77), LL correction angle may have greater practical relevance for intraoperative surgical planning, as it is a parameter that can be directly adjusted during the surgical procedure.

In this study, we assessed the patient’s ability to control their head by active neck flexion and maintain horizontal gaze as the primary outcome at 3 months after NMS correction. The reason for setting the outcome assessment at postoperative 3 months is based on previous observations that sitting balance significantly improves compared to the preoperative state at a minimum of 3 months after NMS correction [21]. We also tried to minimize the influence of NMD progression on the patient’s head control ability by selecting postoperative 3 months as the most appropriate timing of outcome evaluation. After a longer follow-up, it will be difficult to tell whether the loss of head control is due to sagittal balance changes after surgery or the progression of NMD itself.

However, among 31 patients in the headrest-required group, 4 patients were able to regain the ability to maintain independent horizontal gaze without the use of a headrest at postoperative 1 year. Identifying the mechanism and associated factors for this restoration of head control ability is beyond the scope of this study and should be the subject of future research.

Besides the radiological factors described above, no clinical factors were significantly associated with the loss of head control after NMS correction in this study. However, the headrest-required group tended to have more girls than the non-required group (32.2% vs. 15.8%, *p* = 0.128). Among 21 spinal muscular atrophy (SMA) patients in our study, 13 were type 2, and the other 8 were type 3. The patients with type 2 SMA, which is associated with more severe clinical manifestations [22] showed a predominance of female patients (male 3, female 10), whereas the milder type 3 SMA patients had a predominance of male patients (male 6, female 2). Accordingly, the higher proportion of female patients in the headrest-required group is more plausibly attributed to this subtype distribution rather than gender itself serving as an independent risk factor.

This study has several limitations. First, as a single-center retrospective analysis, selection bias may have influenced the choice of surgical intervention. Second, the current study cohort consists of patients with heterogeneous NMD. Therefore, the effect of underlying NMD on the loss of head control cannot be ruled out. Third, we did not perform objective measurements of neck flexor and extensor muscle power in this study. Variations in neck muscle strength may still have influenced the outcomes. Therefore, in future studies, it would be necessary to assess the power of the neck flexor muscles. Despite these limitations, this is the first study to report that head control may be difficult owing to sagittal balance change after spinal deformity correction surgery in patients with NMS.

## Conclusion

In this study, the loss of ability to maintain independent horizontal gaze after deformity correction for NMS was associated with a greater increase in LL and correction of positive sagittal balance. Therefore, avoiding excessive LL restoration and maintaining preoperative sagittal balance during surgical procedures is crucial for preserving head control ability in patients undergoing deformity correction for NMS.

## Data Availability

The datasets used and/or analyzed during the current study are available from the corresponding author on reasonable request.

## Abbreviations

NMS: Neuromuscular scoliosis
NMD: Neuromuscular diseases
CL: Cervical lordosis
TK: Thoracic kyphosis
LL: Lumbar lordosis
SVA: Sagittal vertical axis
T1S: T1 slope
CBVA: Chin-brow vertical angle
OR: Odds ratio
DMD: Duchenne muscular dystrophy
ASD: Adult spinal deformity
ROC: Receiver operating characteristic
AUC: Area under curve
